# Supplementary material for: A Humanized Yeast Phenomic Model of Deoxycytidine Kinase to Predict Genetic Buffering of Nucleoside Analog Cytotoxicity
Source: Genes (Basel). 2019 Sep 30;10(10):770. doi: 10.3390/genes10100770 (PMC6826991; doi:10.3390/genes10100770)
Supplement: Supplementary file 1 [file genes-10-00770-s001.zip › Additional_Files/Additional_File7_GOTermHeatmaps.zip!/Additional_File7_GOTermHeatmaps/regulation_of_transcription_from_RNA_polymerase_I_promoter.pdf]

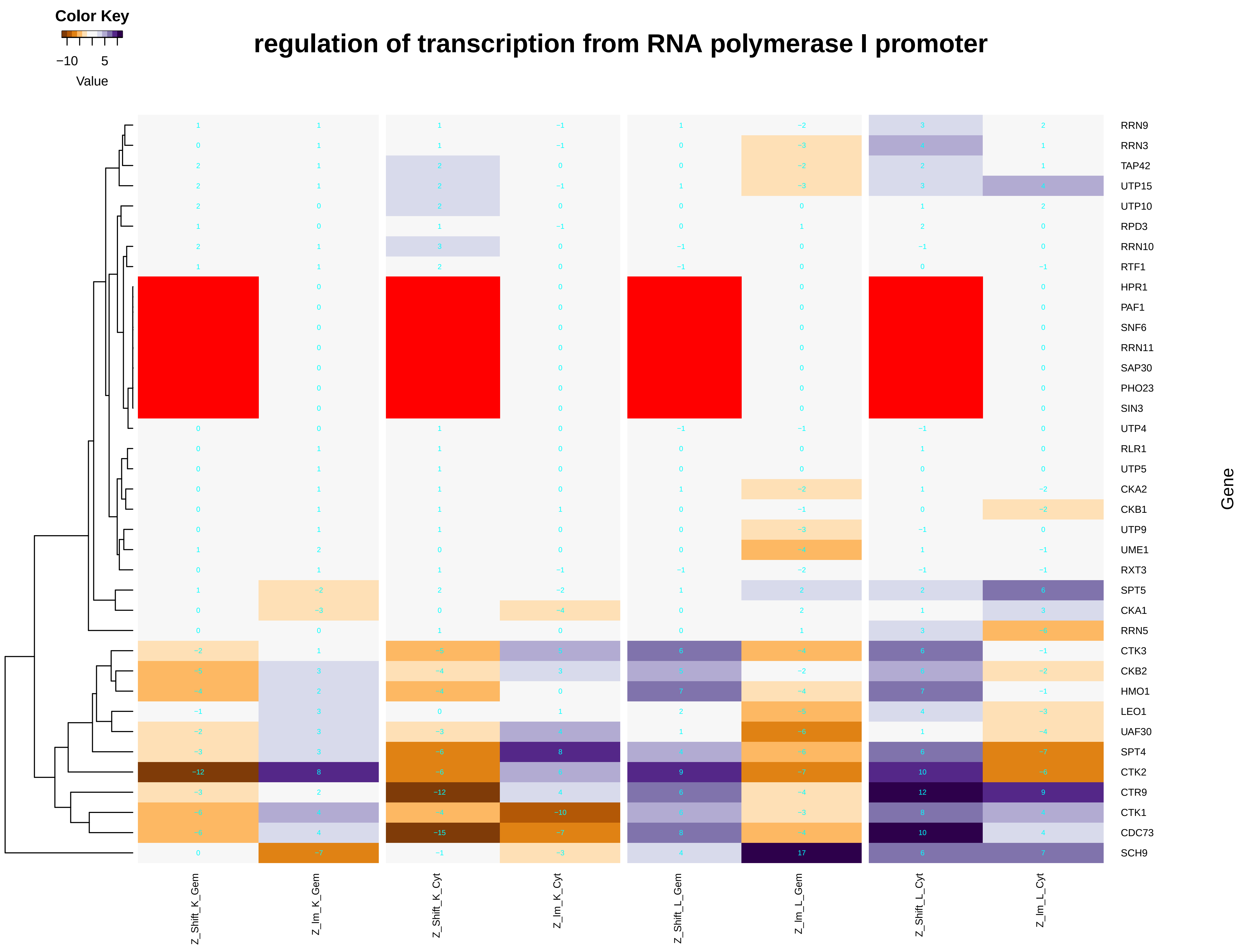
Color Key
−10
5
Value
regulation of transcription from RNA polymerase I promoter
RRN9
RRN3
TAP42
UTP15
UTP10
RPD3
RRN10
RTF1
HPR1
PAF1
SNF6
RRN11
SAP30
PHO23
SIN3
UTP4
RLR1
UTP5
CKA2
CKB1
UTP9
UME1
RXT3
SPT5
CKA1
RRN5
CTK3
CKB2
HMO1
LEO1
UAF30
SPT4
CTK2
CTR9
CTK1
CDC73
SCH9
Gene
Z_Shift_K_Gem
Z_Im_K_Gem
Z_Shift_K_Cyt
Z_Im_K_Cyt
Z_Shift_L_Gem
Z_Im_L_Gem
Z_Shift_L_Cyt
Z_Im_L_Cyt

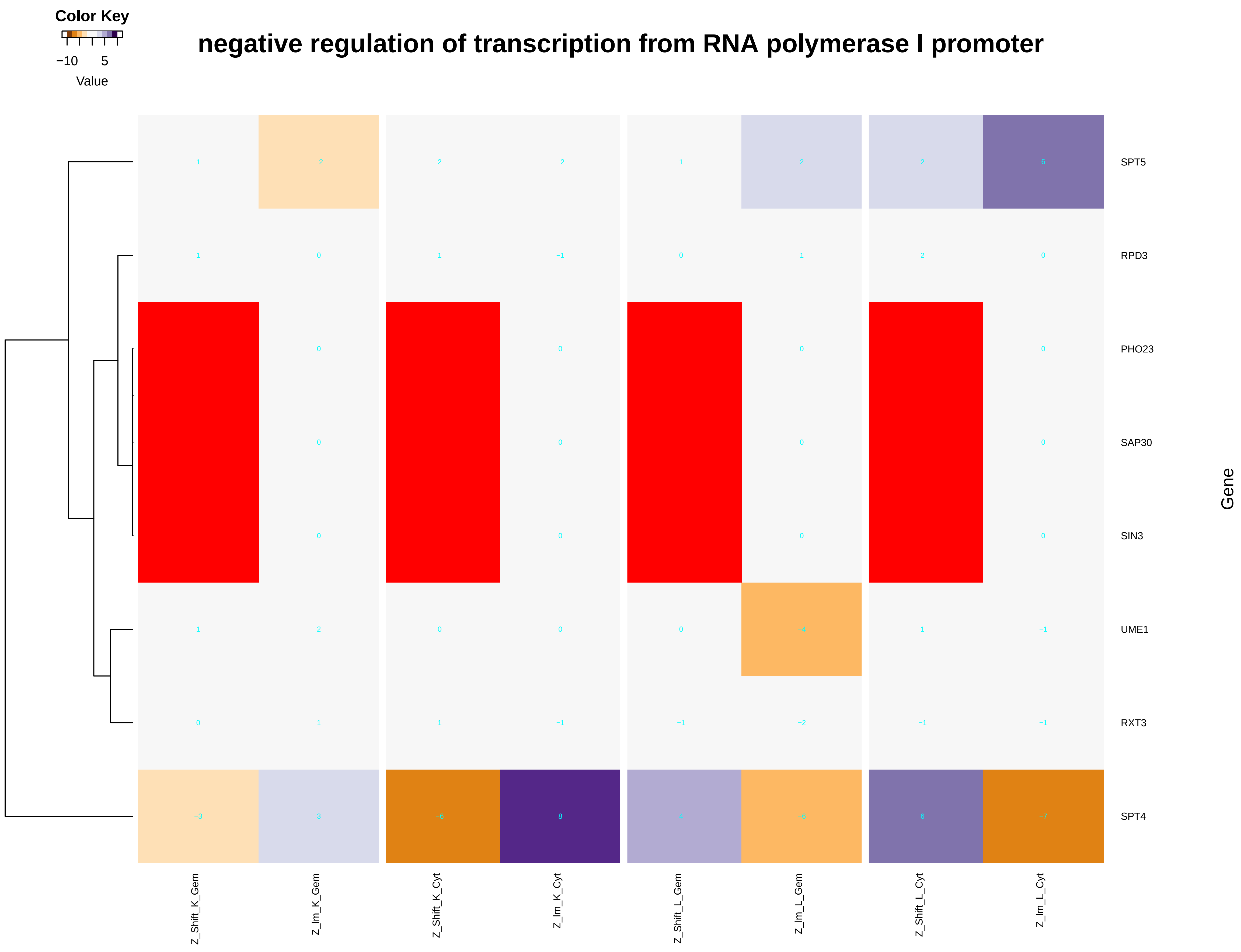
Color Key
−10 5
Value
negative regulation of transcription from RNA polymerase I promoter
SPT5
RPD3
PHO23
SAP30
SIN3
UME1
RXT3
SPT4
Gene
Z_Shift_K_Gem
Z_Im_K_Gem
Z_Shift_K_Cyt
Z_Im_K_Cyt
Z_Shift_L_Gem
Z_Im_L_Gem
Z_Shift_L_Cyt
Z_Im_L_Cyt

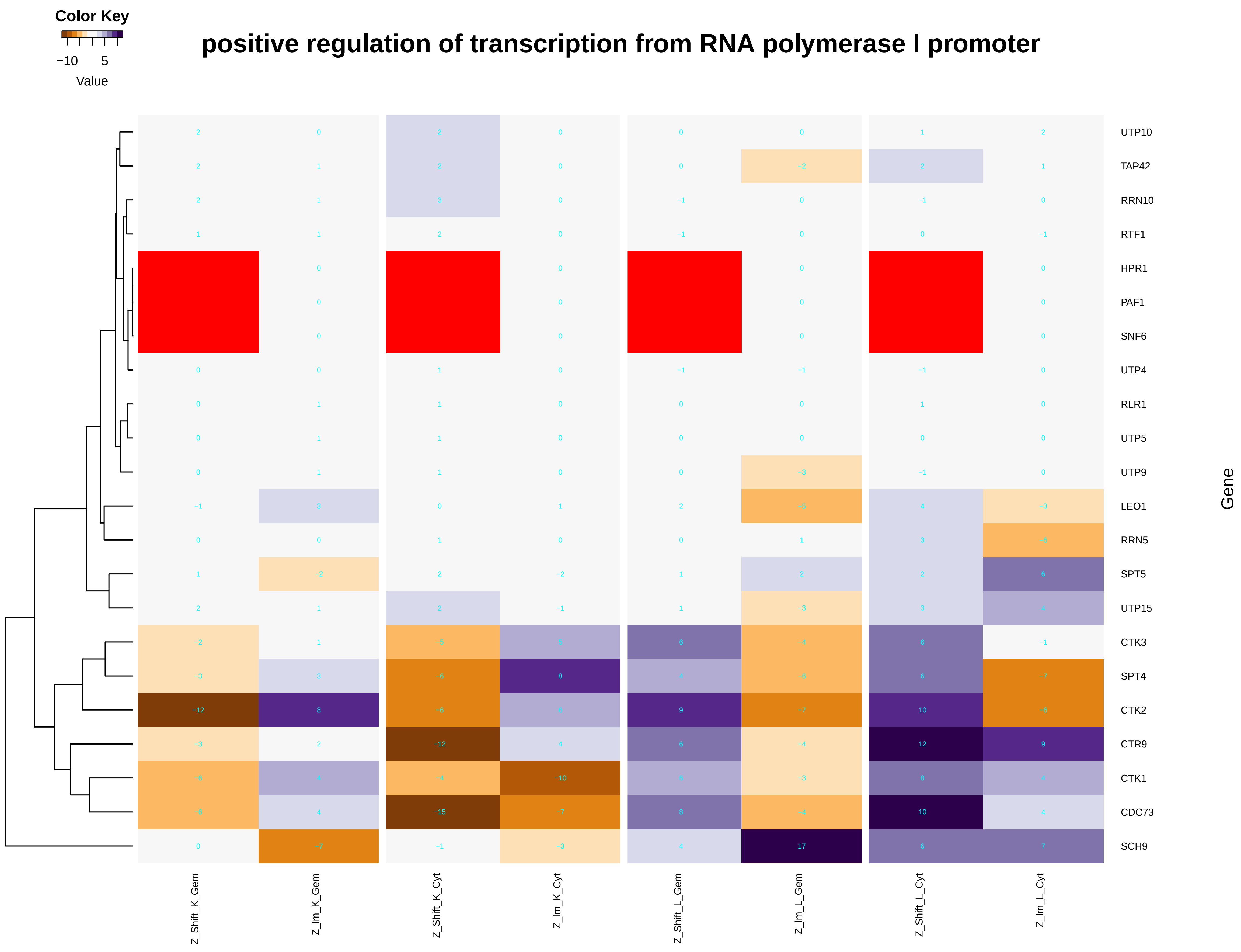

Color Key
−10
5
Value
positive regulation of transcription from RNA polymerase I promoter
UTP10
TAP42
RRN10
RTF1
HPR1
PAF1
SNF6
UTP4
RLR1
UTP5
UTP9
LEO1
RRN5
SPT5
UTP15
CTK3
SPT4
CTK2
CTR9
CTK1
CDC73
SCH9
Gene
Z_Shift_K_Gem
Z_Im_K_Gem
Z_Shift_K_Cyt
Z_Im_K_Cyt
Z_Shift_L_Gem
Z_Im_L_Gem
Z_Shift_L_Cyt
Z_Im_L_Cyt

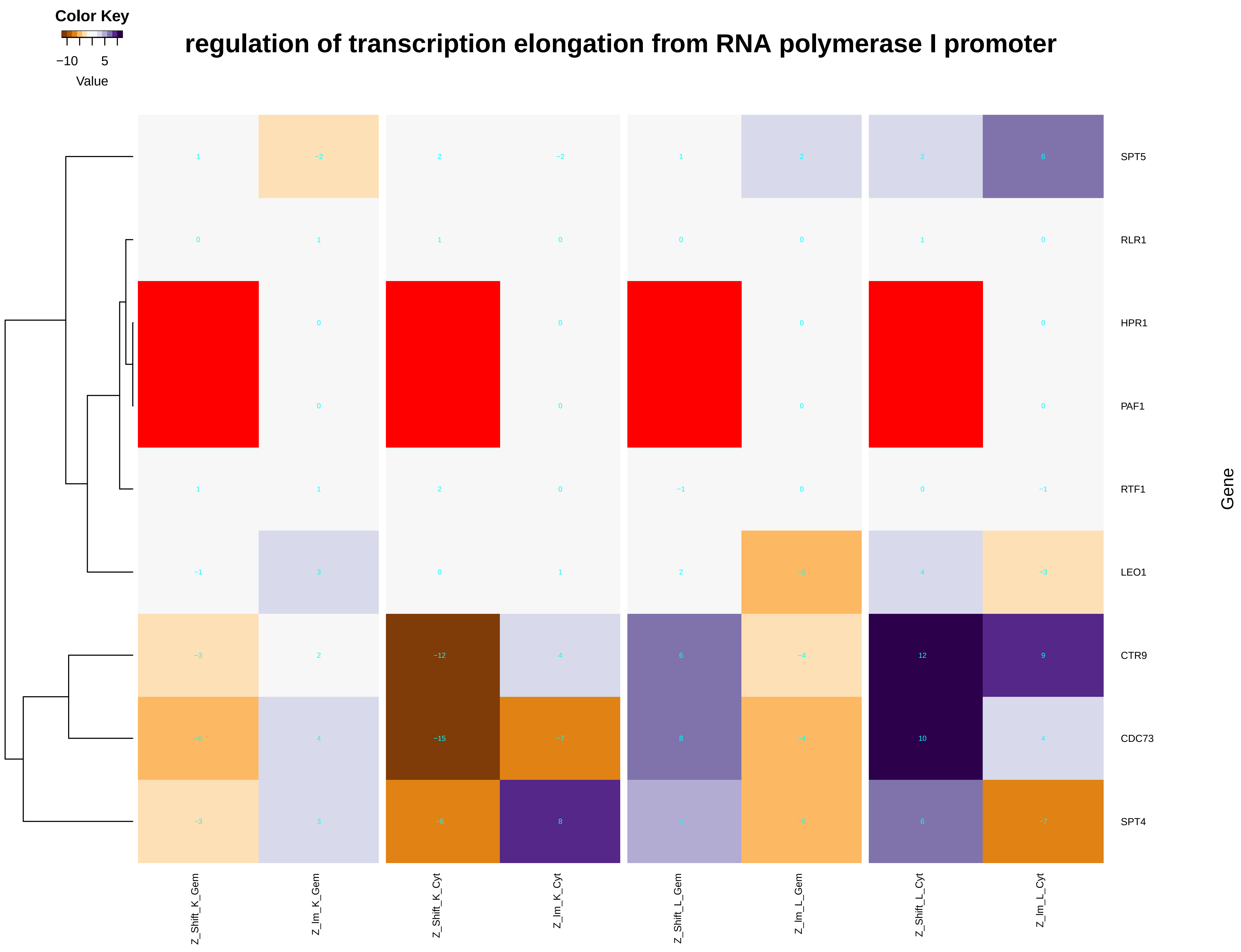
Color Key
−10
5
Value
regulation of transcription elongation from RNA polymerase I promoter
SPT5
RLR1
HPR1
PAF1
RTF1
LEO1
CTR9
CDC73
SPT4
Gene
Z_Shift_K_Gem
Z_Im_K_Gem
Z_Shift_K_Cyt
Z_Im_K_Cyt
Z_Shift_L_Gem
Z_Im_L_Gem
Z_Shift_L_Cyt
Z_Im_L_Cyt

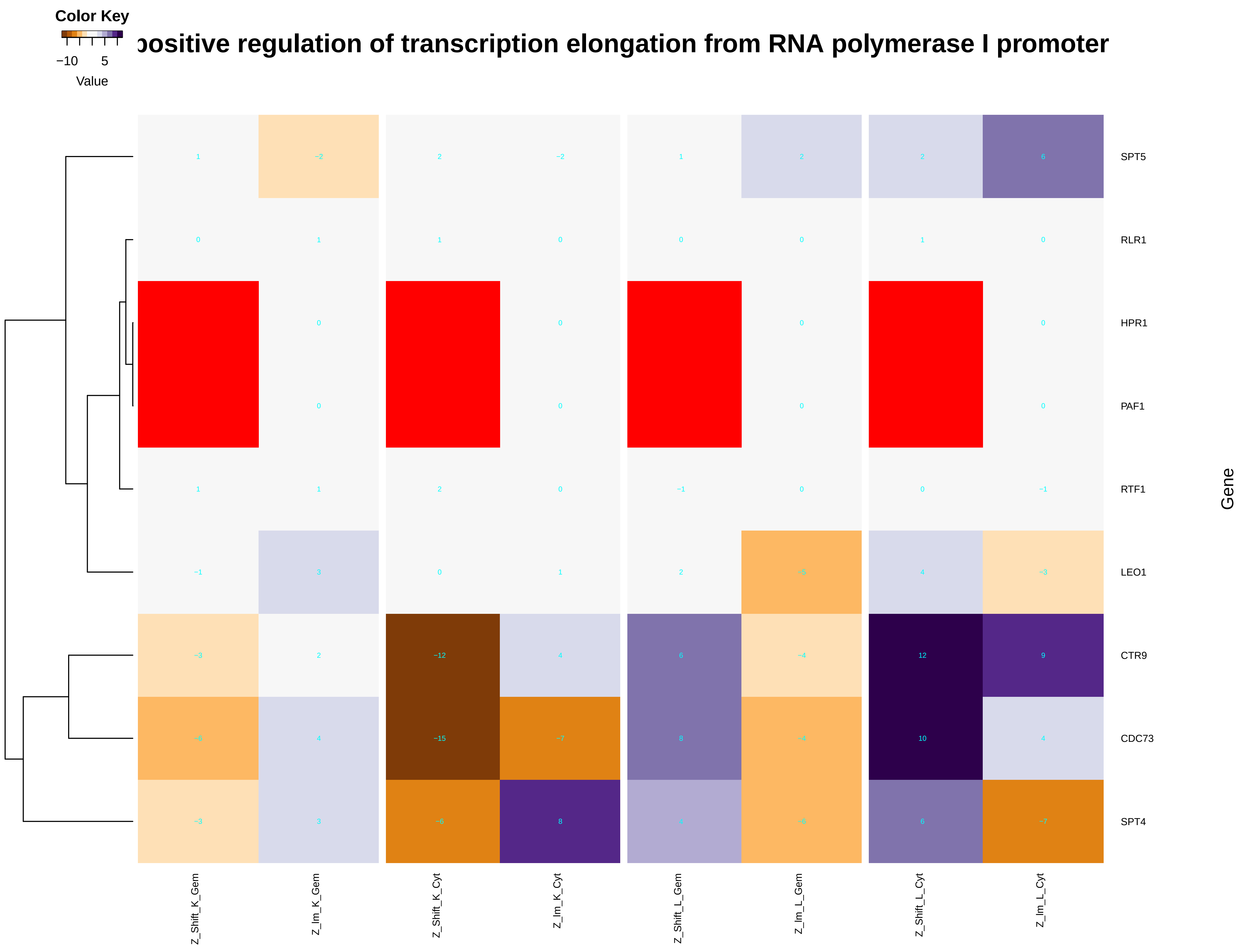
Color Key
−10 5
Value
positive regulation of transcription elongation from RNA polymerase I promoter
SPT5
RLR1
HPR1
PAF1
RTF1
LEO1
CTR9
CDC73
SPT4
Gene
Z_Shift_K_Gem
Z_Im_K_Gem
Z_Shift_K_Cyt
Z_Im_K_Cyt
Z_Shift_L_Gem
Z_Im_L_Gem
Z_Shift_L_Cyt
Z_Im_L_Cyt
